# Effects of voluntary running exercise on bone histology in type 2 diabetic rats

**DOI:** 10.1371/journal.pone.0193068

**Published:** 2018-02-15

**Authors:** Yuri Takamine, Noriko Ichinoseki-Sekine, Takamasa Tsuzuki, Toshinori Yoshihara, Hisashi Naito

**Affiliations:** 1 Graduate School of Health and Sports Science, Juntendo University, Inzai, Chiba, Japan; 2 Faculty of Liberal Arts, The Open University of Japan, Chiba, Chiba, Japan; Indiana University Purdue University at Indianapolis, UNITED STATES

## Abstract

The incidence of obesity in children and adolescents, which may lead to type 2 diabetes, is increasing. Exercise is recommended to prevent and improve diabetes. However, little is known about the bone marrow environment at the onset of diabetes in the young, and it is unclear whether exercise training is useful for maintaining bone homeostasis, such as mechanical and histological properties. Thus, this study clarified the histological properties of bone and whether exercise contributes to maintaining bone homeostasis at the onset of type 2 diabetes in rats. Four-week-old male Otsuka Long-Evans Tokushima Fatty (OLETF; n = 21) rats as a diabetic model and Long-Evans Tokushima Otsuka (LETO; n = 18) rats as a control were assigned randomly to four groups: the OLETF sedentary group (O-Sed; n = 11), OLETF exercise group (O-Ex; n = 10), LETO sedentary group (L-Sed; n = 9), and LETO exercise group (L-Ex; n = 9). All rats in the exercise group were allowed free access to a steel running wheel for 20 weeks (5–25 weeks of age). In the glucose tolerance test, blood glucose level was higher in the O-Sed group than that in the L-Sed and L-Ex groups, and was markedly suppressed by the voluntary running exercise of O-Ex rats. The energy to fracture and the two-dimensional bone volume at 25 weeks of age did not differ significantly among the groups, though the maximum breaking force and stiffness were lower in OLETF rats. However, bone marrow fat volume was greater in O-Sed than that in L-Sed and L-Ex rats, and was markedly suppressed by wheel running in the O-Ex rats. Our results indicate that exercise has beneficial effects not only for preventing diabetes but also on normal bone remodeling at an early age.

## Introduction

Diabetes mellitus, especially type 2 diabetes, is increasing worldwide [1]. Diabetics are at risk of complications such as diabetic retinopathy, nephropathy, and neuropathy, which may reduce their quality of life. Currently, the incidence of obesity in children and adolescents is increasing, which may lead to type 2 diabetes. Poor fitness habits or excessive food intake during youth may be related to the increasing incidence of diabetes.

During the growth period, bone modeling and remodeling serve to maintain bone homeostasis. However, little is known of the histological and mechanical characteristics of bone in early life and how they are related to those of bone at the onset of diabetes. Bone fragility in diabetic patients has attracted attention [2–4], and several studies have suggested that elderly patients are at risk of diabetes-related fractures [5–7]. A meta-analysis showed that men and women with type 2 diabetes have a high hip fracture risk, which is thought to be due to diabetic complications, such as diabetic retinopathy [8]. The strength of bone depends on both the bone mineral density (BMD) and bone structure. Interestingly, however, Vestergaard *et al*. [7] showed that type 2 diabetics have a higher risk of hip fracture than non-diabetics, despite their high BMD. They also suggested that the fracture risk in diabetes depends on bone structure rather than BMD, indicating that bone structure is affected by the bone marrow environment [9]. Again, little is known of the bone marrow environment at the onset of diabetes in young people.

There are several approaches for preventing diabetes. Exercise has been recommended to prevent and improve diabetes [10] because long-term endurance and resistance training can improve insulin resistance [11]. Nevertheless, endurance training is prescribed mainly for type 2 diabetes. The American College of Sports Medicine has recommended treadmill running as an aerobic exercise to prevent and treat diabetes [12]. Indeed, Kasumov *et al*. [13] showed that treadmill running intervention for 12 weeks improved insulin resistance in humans. Experiments using laboratory animals have also shown that endurance training on a treadmill or wheel was useful for treating diabetes [14–16]. However, it is unclear whether such exercise training is useful for maintaining bone homeostasis (not only compact bone but also bone marrow). Therefore, this study clarified the histological properties of bone and whether exercise contributes to maintaining bone homeostasis at the onset of disease in type 2 diabetic rats.

## Materials and methods

### Animals

Four-week-old male Otsuka Long-Evans Tokushima Fatty (OLETF; n = 21) rats as a diabetic model and Long-Evans Tokushima Otsuka (LETO; n = 18) rats as a control were purchased from Japan SLC (Hamamatsu, Shizuoka, Japan). OLETF rats develop hyperglycemia after 18 weeks of age, and most OLETF rats have diabetes at 25 weeks of age [15, 17]. Our group also previously confirmed that OLETF rats develop diabetes at 25 weeks of age [18]. At 5 weeks of age, the rats were assigned randomly to four groups: OLETF sedentary group (O-Sed; n = 11), OLETF exercise group (O-Ex; n = 10), LETO sedentary group (L-Sed; n = 9), and LETO exercise group (L-Ex; n = 9). All rats were housed for 20 weeks at 23±1°C on a 12 h:12 h light-dark cycle, and provided with standard diet and water *ad libitum*. All animal experiments in this study were approved by the Juntendo University Animal Care Committee.

### Voluntary wheel running exercise

The rats in the exercise group were allowed free access to a steel running wheel for 20 weeks. Each wheel was connected to a counter to measure running activity. The running distance was calculated by multiplying the number of revolutions by the wheel circumference (1 m). The running wheel was locked for 24 h before the animals were sacrificed.

### Tissue collection and preparation

All rats were sacrificed at 25 weeks of age. Blood samples were obtained from the inferior vena cava under anesthesia after fasting overnight and centrifuged at 4°C and 3000 rpm for 10 min to separate serum and plasma. White adipose tissue was removed from the periphery of the epididymis, and the samples were stored at –80°C. The right femur was harvested, connective tissue was removed, and maximum breaking force, stiffness, energy to fracture was measured immediately. Then, the distal metaphysis of each femur was cut off and fixed in 4% paraformaldehyde buffer at 4°C for about 4 days for histological measurements. The fixed sample was washed with water, dehydrated, and decalcified with 10% ethylenediaminetetraacetic acid buffer at 4°C for about 2 weeks. The decalcified sample was embedded in paraffin.

### Blood analysis

Blood glucose levels were measured using the Glutest Neo Super device (Sanwa Kagaku Kenkyusho, Aichi, Japan). Plasma insulin concentrations were determined using an AKRIN-010S rat insulin ELISA kit (Shibayagi, Gunma, Japan). Serum adiponectin and leptin levels were analyzed using enzyme-linked immunosorbent assays (Shibayagi).

### Testing bone mechanical properties

The bone mechanical strength of the right femur at mid-shaft was assessed in a three-point bending test [19]. Connective tissue was removed, and the bone was mounted on two support stands 10.6 mm apart. Then, the crosshead was applied to the metaphysis of the femur, which was compressed by the crosshead at a speed of 2.0 mm/min until fracture occurred. The maximum breaking force was divided by body weight.

### Histomorphometry

Tissue specimens were sectioned at 4 μm thicknesses and stained with hematoxylin and eosin to assess bone histology. A qualitative histological analysis was performed as described previously. Briefly, the Bone area/Tissue area (B.Ar/T.Ar; %) as a static measurement of bone was calculated for the secondary spongiosa of the distal femoral metaphysis. The Adipocyte area/Tissue area (Ad.Ar/T.Ar; %) was used as a measure of bone marrow fat. Adipocytes were identified morphologically as described previously [20]. The area examined was located 1 mm distal to the growth plate, and measured 1.6 mm long and 2.0 mm wide in the direction of the long axis. All quantitative analyses of microscopic observations were performed using image analysis software (KS400; Carl Zeiss AG, Oberkochen, Germany).

### Statistical analysis

All data are presented as the mean±standard error; statistical significance was accepted at *p*<0.05. A two-way analysis of variance was used for the statistical analysis. If the significance level was reached, the comparison was made using Bonferroni’s *post hoc* test.

## Results

### Running distance, body weight, and white adipose tissue weight

The total distance run was 684±88 and 578±39 km in the L-Ex and O-Ex groups, respectively; the difference was not significant. At the beginning of the experiment (5 weeks of age), body weight did not differ among the groups. On the other hand, at 25 weeks of age, the O-Sed group was heavier than the L-Sed group, while no difference was observed between the exercise groups (Table 1). Similarly, the white adipose tissue weight was higher in O-Sed than in L-Sed at 25 weeks of age, while this difference was attenuated by the wheel running. Food consumption was higher in O-Sed than in L-Sed from 15 to 25 weeks of age (*p*<0.05), demonstrating overeating by the OLETF rats. No significant difference was observed in food consumption between the L-Ex and O-Ex groups.

**Table 1:** Body weight, food consumption and white adipose tissue weight.

	L-Sed (n = 9)	L-Ex (n = 9)	O-Sed (n = 11)	O-Ex (n = 10)
Body weight at 5wk (g)	103.3 ± 2.6	107.4 ± 1.3	124.0 ± 2.6	126.8 ± 2.6
Body weight at 25wk (g)	482.5 ± 9.0	436.0 ± 7.3^†^	625.6 ±10.1*	452.3 ± 12.9
Food consumption (g/day)	30.1 ± 0.5	35.7 ± 1.3	42.2 ± 0.7^†^	38.5 ± 1.0
White adipose tissue (g)	8.1 ± 0.6	5.1 ± 0.4^†^	15.7 ± 0.9*	4.2 ± 0.4

^†^; p <0.05 versus L-Sed

*; p<0.05 versus all

### Blood analysis related to diabetes

**Fig 1:**
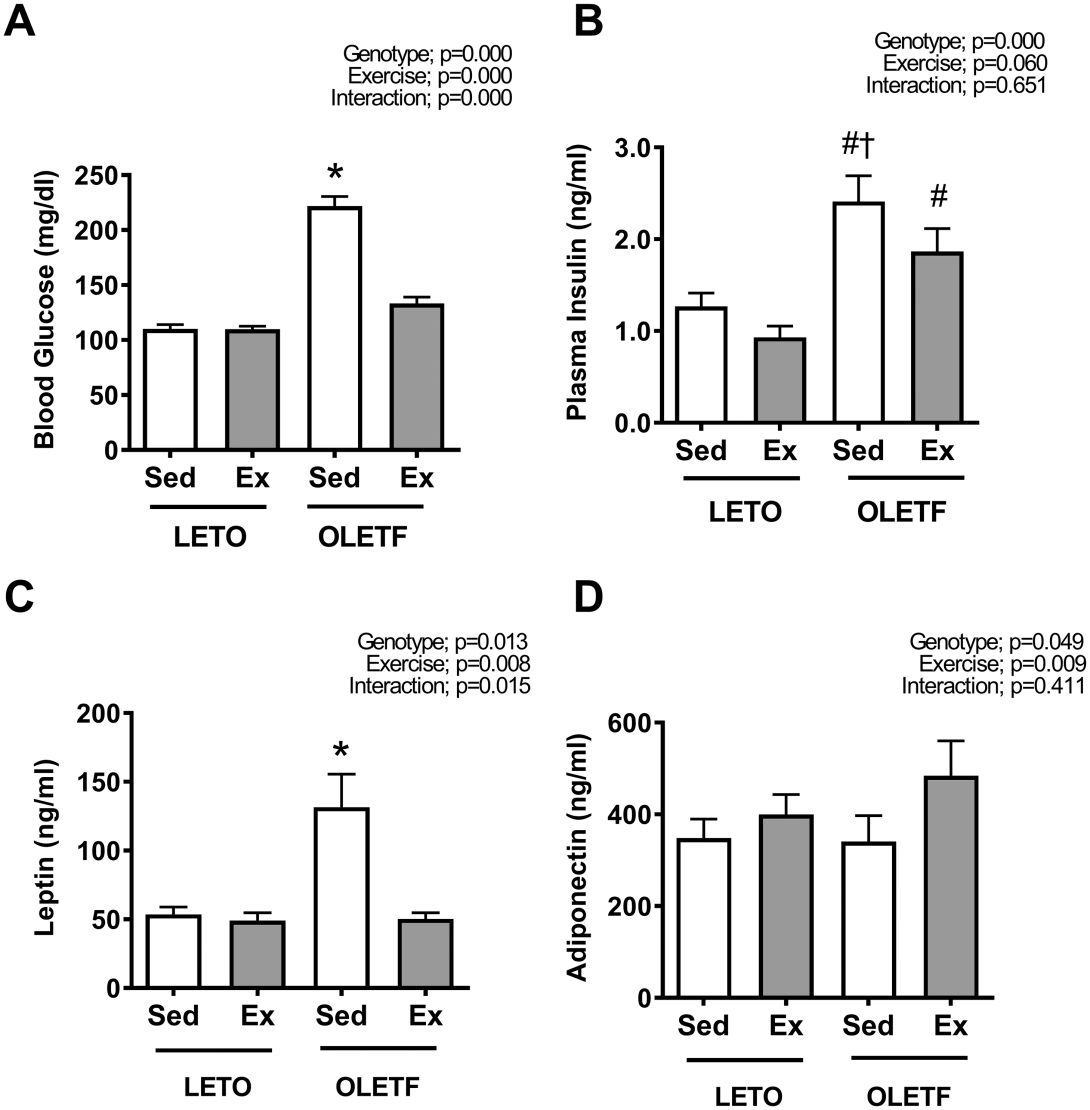
Blood analysis related to diabetes. The blood glucose (A), plasma insulin (B) and serum leptin (C) levels were significantly greater in O-sed rats than in the LETO rats, but it was attenuated by exercise. Serum adiponectin (D) levels were increased by exercise. All values are presented as the mean ± SEM. * *p*<0.05 vs. all; † *p*<0.05 vs. L-Sed; # *p*<0.05 vs. L-Ex.

Blood glucose levels were significantly greater in the OLETF rats than those in LETO rats (*p*<0.05). Exercise decreased the fasting blood glucose level in the O-Ex group to the values observed in LETO rats (Fig 1A, *p*<0.05). Exercise also tended to decrease fasting plasma insulin levels in O-Ex rats, but there was no statistical significance (Fig 1B). The serum leptin level was highest in the O-Sed group (*p*<0.05), and exercise prevented elevated leptin levels (Fig 1C). Serum adiponectin levels were increased by exercise (Fig 1D).

### Bone mechanical properties

**Fig 2:**
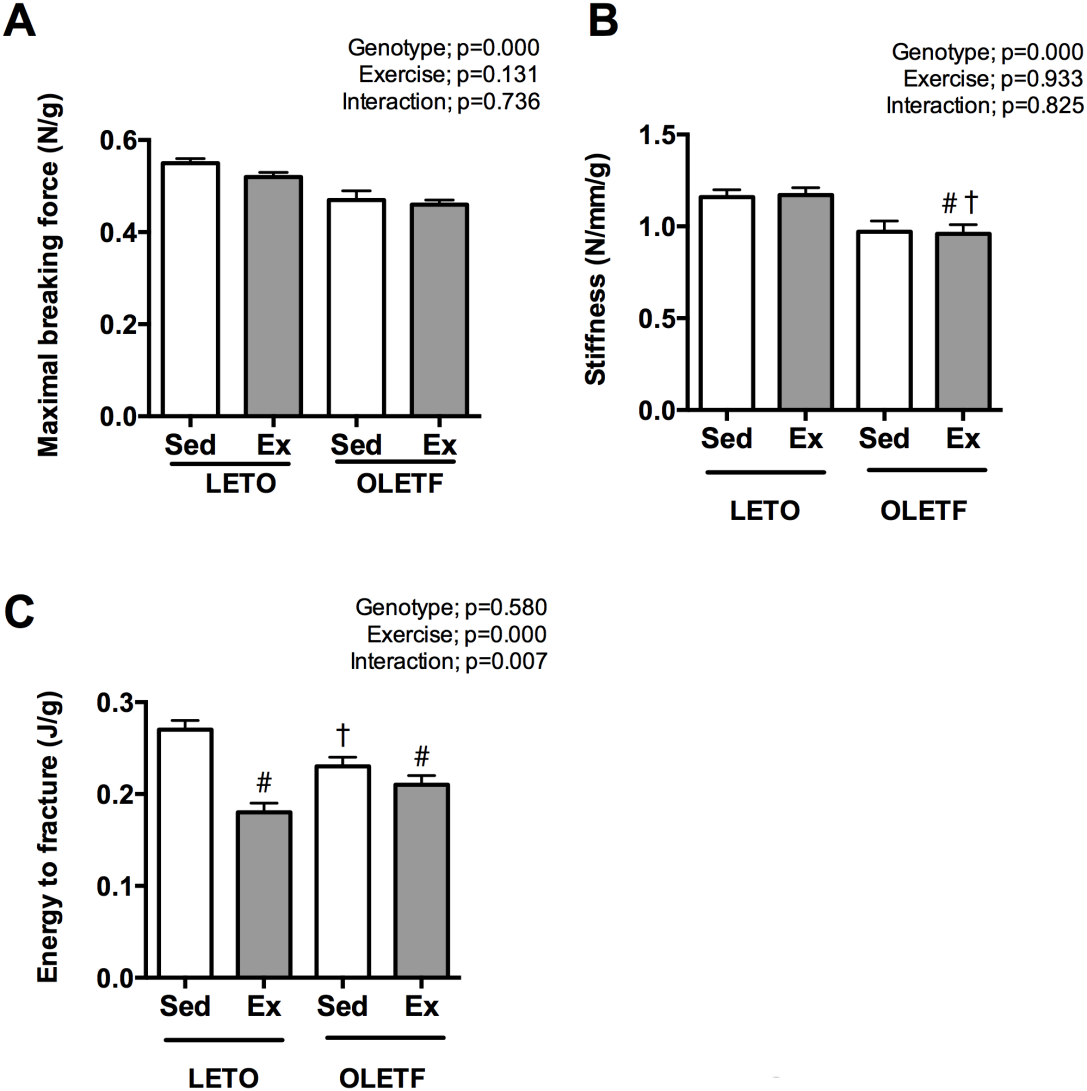
The bone mechanical properties of the rats at 25 weeks of age. Maximum breaking force (A) and stiffness (B) were lower in OLETF rats. In contrast, energy to fracture was not different between genotype. All values are presented as the mean ± SEM. * *p*<0.05 vs. all; † *p*<0.05 vs. L-Sed; # *p*<0.05 vs. L-Ex.

Maximum breaking force (Fig 2A) and stiffness (Fig 2B) of the femur at 25 weeks of age were lower in OLETF rats (*p*<0.05). On the other hand on the energy to fracture (Fig 2C), no significant difference was observed between LETO and OLETF rats but it was decreased by exercise (*p*<0.05).

### Bone histological properties

**Fig 3:**
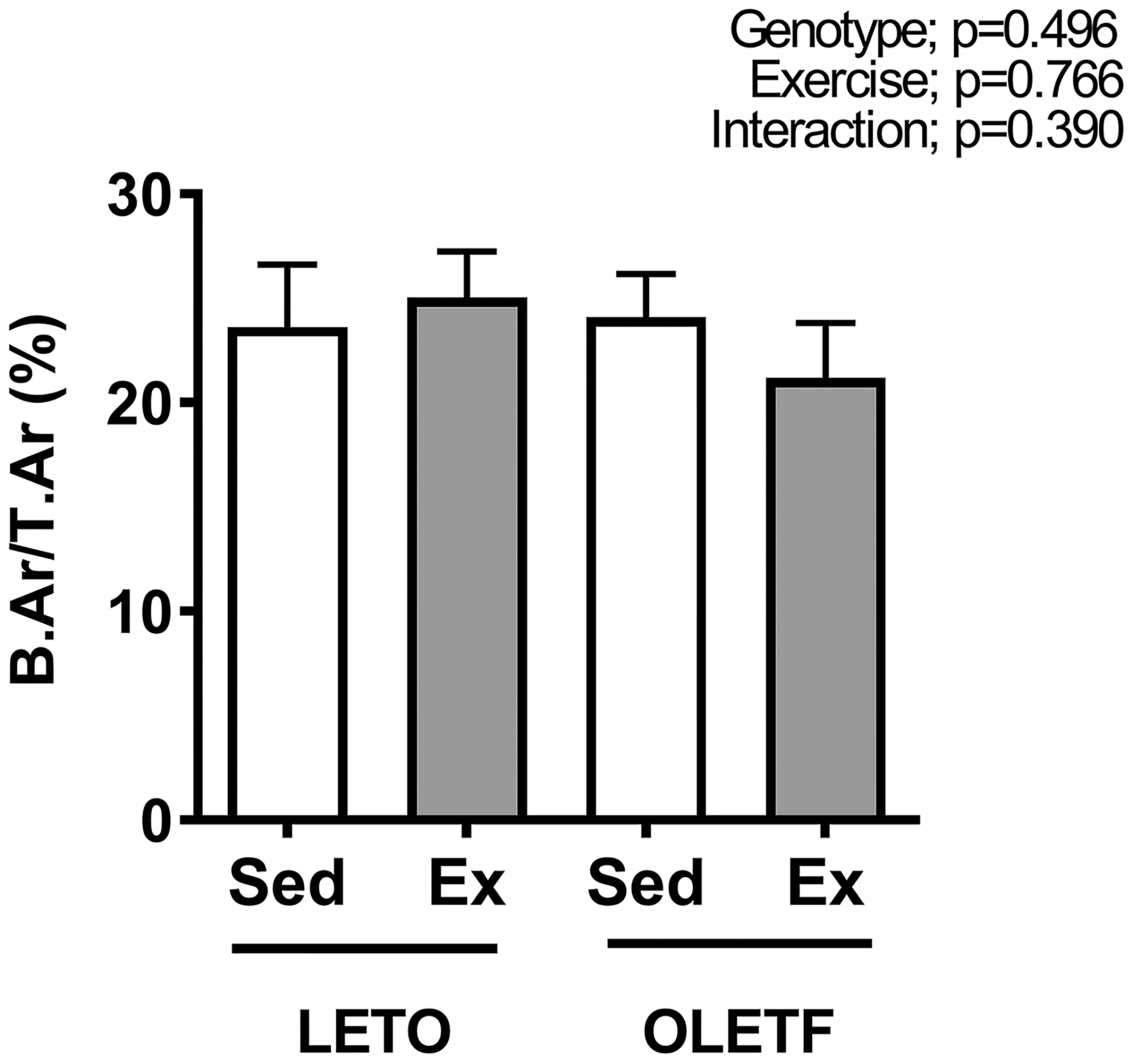
The bone volume at the distal end of the femur as assessed by two-dimensional histomorphometry at 25 weeks of age. All values are presented as the mean ± SEM.

The two-dimensional bone volume (B.Ar/T.Ar) at the distal end of the femur measured histologically at 25 weeks of age did not differ significantly among the groups (Fig 3).

### Bone marrow fat

**Fig 4:**
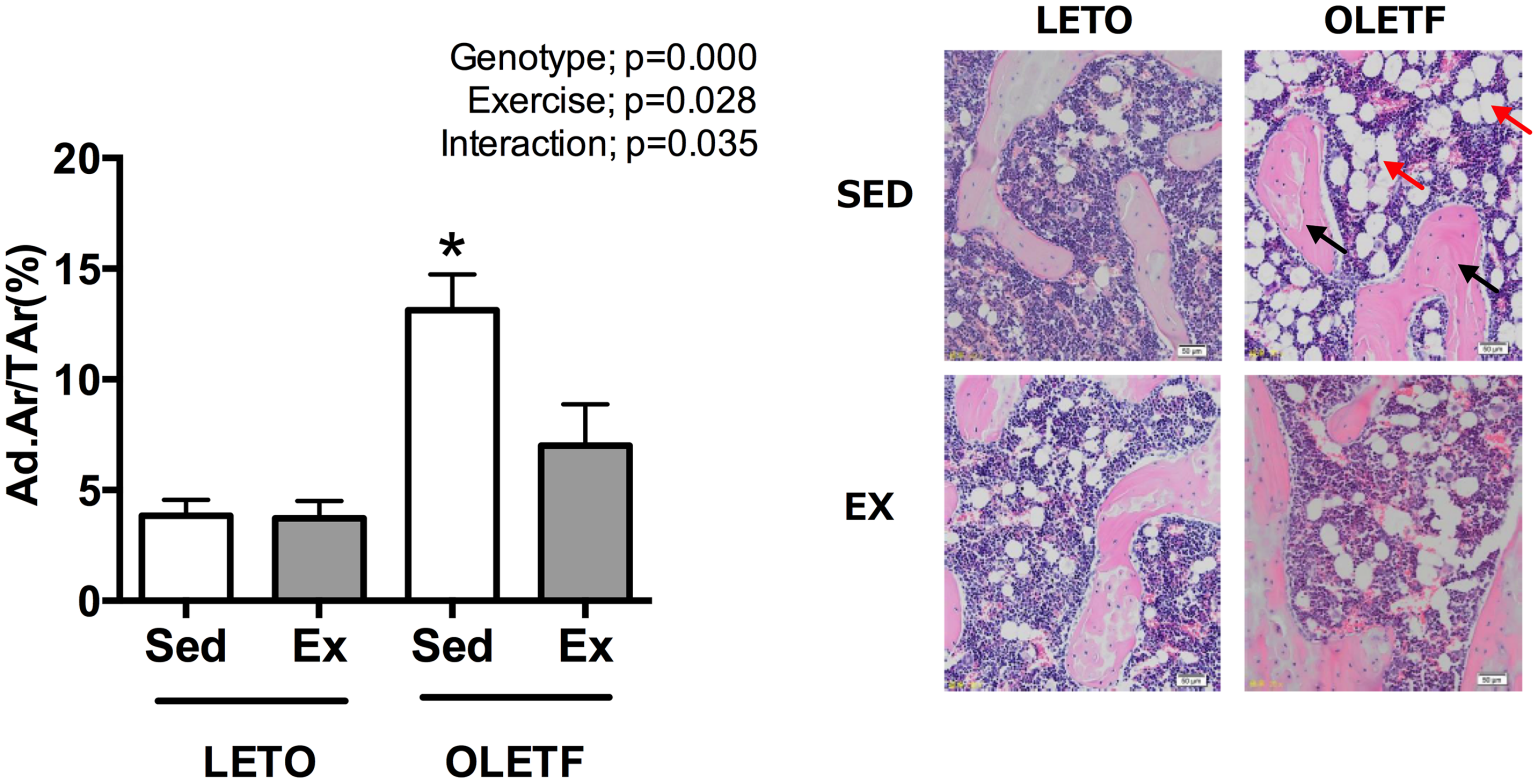
The marrow fat volume at the distal end of the femur as assessed by two-dimensional histomorphometry (A) and a micrograph of marrow fat (B) at 25 weeks of age. The black arrow indicates bone tissue, and red arrow shows marrow fat in micrograph. All values are presented as the mean ± SEM. * *p*<0.05 vs. all; † *p*<0.05 vs. L-Sed; # *p*<0.05 vs. L-Ex.

The bone marrow fat volume (Ad.Ar/T.Ar) at the distal end of the femur assessed using two-dimensional histomorphometry was greater in O-Sed rats than in L-Sed and L-Ex, and was markedly suppressed by wheel running in the O-Ex rats (Fig 4).

## Discussion

Obese and diabetic patients have a high fracture risk [21]. The increased fracture risk in diabetes may be due to increasing bone fragility caused by diabetes or an increased incidence of falling, which is related to diabetic complications [22]. Several studies have suggested that bone fragility in diabetes is caused by adiposity, insulin resistance, fatty acid composition, and hormones [23–24]. Marrow fat may negatively affect bone fragility in diabetes. The accumulation of marrow fat affects the balance of bone turnover, which may lead to a decrease in bone volume or bone quality [25].

In this study, we wanted to clarify the bone histology at the onset of type 2 diabetes and whether exercise, which prevents the onset of diabetes, can maintain bone homeostasis. OLETF rats were used as a model of diabetes because they exhibit similar clinical and pathological features to humans with non-insulin-dependent diabetes mellitus [17]. We previously reported that OLETF rats have the features of type 2 diabetes at 25 weeks of age; the abnormal glucose tolerance in the OLETF rats may be due to insulin resistance because no impairment of insulin secretion was observed [18]. However, the symptoms of type 2 diabetes were prevented by voluntary exercise at an early age [18].

At 25 weeks of age, the maximum breaking force of bone, energy to fracture, and two-dimensional bone volume (%, B.Ar/T.Ar) were not affected by the onset of diabetes. On the other hand, bone stiffness was lower in OLETF rats as same as previous study [26]. Fajardo *et al*. [25] reviewed studies of type 2 diabetic bone fragility in a rodent model and proposed that bone fragility in diabetes is dependent on the age of onset; bone fragility was observed after onset of the disease. As in Fajardo *et al*. [27], the two-dimensional bone volume in OLETF rats was not affected by the onset of diabetes in our study, possibly due to the early stage of bone development. However, Hinton *et al*. [28] reported that 13-week-old pre-diabetic OLETF rats had a low BMD and reductions in bone formation markers, suggesting that the harmful influence of diabetes on bone had already begun before the onset of diabetes.

We found that the bone marrow fat volume was increased at the onset of diabetes in OLETF rats compared to LETO rats. It is known that bone marrow fat volume increases with aging or obesity. Styner *et al*. [9] suggested that bone marrow fat is an energy storage depot, and they confirmed that the increase in bone marrow fat induced by a high-fat diet was at least as great as the increase in perigonadal white adipose tissue. Indeed, in our study, both bone marrow fat and perigonadal white adipose tissue were higher in the OLETF rats at 25 weeks of age. However, little is known about the mechanism underlying the accumulation of adipocytes in bone marrow. Interestingly, the accumulation of fat or a change in the fatty acid composition in bone marrow may cause the bone loss seen in elderly, obese, or diabetic individuals [24, 25, 29, 30]. In particular, elderly diabetic patients had more saturated bone marrow fat, and their BMD was low compared with non-diabetics [30]. Similarly, Halade *et al*. [31] reported that obesity in mice induced by a high-fat diet for 6 months resulted in bone loss and marrow adiposity. In the current study, however, an increase in bone marrow fat was observed but no decrease was seen in the two-dimensional bone volume in OLETF rats. Further study is needed to clarify the mechanisms of the accumulation of bone marrow fat and bone loss.

We also demonstrated the effects of voluntary exercise on the onset of diabetes and bone marrow adiposity. The total running distance in our study was similar to that in other studies [32], and the abnormal glucose tolerance in OLETF rats was prevented by exercise. Our results support a previous study that showed that voluntary running exercise was effective at improving insulin sensitivity and glucose uptake [14]. Moreover, in our study, voluntary exercise decreased the increase in bone marrow fat observed in OLETF rats. Many reports have indicated that exercise has the potential to inhibit the accumulation of marrow fat [9, 33], which could be beneficial for normal bone remodeling. Similarly, Styner *et al*. [29] reported that the increase in bone marrow fat induced by a high-fat diet was reduced by voluntary running for 6 weeks in 8-week-old female mice. Yuki *et al*. [33] suggested that jumping exercise decreased the marrow fat volume and increased osteogenic factors via mechanical loading. Minematsu *et al*. [34] and Hinton *et al*. [28] showed a beneficial effect of wheel running on bone properties, such as geometry and mechanical strength, in OLETF rats. However, in our study, maximum breaking force of bone and two-dimensional bone volume were not affected by voluntary running. One reason for this difference may be the duration of the exercise period. In our study, the wheel-running period was shorter (5 months) than those of previous reports (17 months and 9 months). Another possibility is the bone property evaluation technique; the resolution of micro computed tomography analysis is higher than histology. Our results show a positive effect of voluntary exercise on bone marrow fat, but these two factors may mask the beneficial effects of exercise on bone properties; further investigation is needed for clarification.

Leptin is involved in bone formation and resorption via direct and indirect pathways [35]. However, Caro *et al*. [36**]** demonstrated that obese subjects have leptin resistance; consequently, the circulating leptin level was not useful for explaining the involvement of bone metabolism. The effect of exercise on the serum leptin level in OLETF rats may affect the white adipose tissue volume. Nevertheless, we observed no difference in serum adiponectin levels by genotype while it was increased by exercise. Adiponectin has both positive and negative effects on bone formation via distinct pathways [37], and it inversely related to insulin resistance [38, 39]. Further study is needed to clarify the relationship between those adipocytokines and bone at the onset of diabetes.

## Conclusions

In conclusion, our results demonstrate that the onset of type 2 diabetes did not affect bone volume, while marrow fat accumulated. This accumulation of bone marrow fat was inhibited by 20 weeks of voluntary running exercise, which also prevented the onset of diabetes in OLETF rats, suggesting that exercise has beneficial effects not only on the prevention of diabetes but also on normal bone remodeling at an early age.
